# Metabolic profile response to administration of epigallocatechin-3-gallate in high-fat-fed mice

**DOI:** 10.1186/1758-5996-6-84

**Published:** 2014-08-12

**Authors:** Mayara Franzoi Moreno, Rachel De Laquila, Marcos Hiromu Okuda, Fábio Santos Lira, Gabriel Inácio de Morais Honorato de Souza, Cláudio Teodoro de Souza, Monica Marques Telles, Eliane Beraldi Ribeiro, Claudia Maria Oller do Nascimento, Lila Missae Oyama

**Affiliations:** Departamento de Fisiologia, Universidade Federal de São Paulo-EPM, São Paulo, SP Brasil; Faculdades Integradas Coração de Jesus – FAINC, Santo André, SP Brasil; Immunometabolism Research Group, Department of Physical Education, Universidade Estadual Paulista, UNESP, Presidente Prudente, SP Brazil; Laboratory of Exercise Biochemistry and Physiology, Health Sciences Unit, University of Southern Santa Catarina, Criciúma, SC Brazil; Departamento de Ciências Biológicas, Universidade Federal de São Paulo-Campus Diadema, Diadema, SP Brasil

**Keywords:** High-fat diet, Adipokines, Adipose tissue, Inflammation

## Abstract

**Background:**

Obesity is associated with increased adipose tissue and glucose intolerance. High-fat diets (HFDs) are known to induce obesity and increase proinflammatory adipokines. The consumption of green tea may improve the health of obese individuals because it contains a potent antioxidant that has effects on body weight, energy expenditure and serum cholesterol concentrations.

**Methods:**

We examined the effects of epigallocatechin-3-gallate (EGCG) (50 mg/kg body weight per day) or saline after 30 or 60 days of treatment. Mice were distributed into four groups: 1) NS: normolipidic diet receiving saline; 2) NE: normolipidic diet receiving EGCG; 3) HFS: high-fat diet receiving saline; 4) HFE: high-fat diet receiving EGCG.

**Results:**

We observed that administration of a HFD plus EGCG treatment for 60 days reduced delta weight, the relative weights of the mesenteric adipose tissue (MES), retroperitonial adipose tissue (RET), epididymal adipose tissue (EPI), the sum of the adipose tissues (SAT), reduced triacylglycerol (TG) and improved both high-density lipoprotein (HDL) cholesterol levels and the adiponectin/STA ratio when compared with HFS.

**Conclusions:**

Our results suggest that the chronic administration of EGCG (60 days) promoted a significant improvement in glucose tolerance, decreased adipose tissue deposits, weight mass, TG and HDL-C only when associated with high-fat diet treatment.

## Introduction

Obesity is characterised by excessive fat accumulation and a resultant imbalance in energy intake and expenditure. Notably, HFD is the mainstream food habit of modern society and is one of the major factors contributing to obesity [1]. The obese state is characterised by low-grade systemic inflammation, primarily resulting from increased adipocyte size and the recruitment of macrophages into the white adipose tissue (WAT) [2]. Adipose tissue is an endocrine organ that releases protein factors, known as adipokines which include hormones implicated in energy balance (e.g. leptin and adiponectin), glucose tolerance and insulin sensitivity (adiponectin and resistin) and classical cytokines (e.g. TNF-α, interleukin-6 and 10) [3, 4].

Several strategies have been adopted for treatment and prevention of obesity, as well as for associated conditions, such as insulin resistance, inflammation and hypertension. Nutritional interventions, including calorie restriction, vitamin supplementation, fresh green tea or extract consumption are often utilised to promote weight loss [2, 5–7].

Green tea (*Camellia sinensis*) is one of the most widely consumed beverages in the world and experimental studies indicate that the use of green tea extract is useful for obesity treatment and prevention [6, 8, 9]. Among the many polyphenols present in green tea, epigallocatechin-3-gallate (EGCG) is the most active form. It is suggested that the action of EGCG occurs in the sympathetic nervous system, specifically by inhibiting the enzyme catechol-O-methyl transferase, which degrades norepinephrine. Thus, EGCG could exert regulatory functions on sympathetic activation and lypolysis. Furthermore, in animal studies, we observed a reduction of blood glucose and insulin after the consumption of green tea [10].

Friedrich *et al*. [11] showed that the anti-obesity effects of EGCG can be explained by decreased food digestibility and nutrient absorption, ultimately resulting in increased post-prandial fat oxidation and reduced incorporation of dietary lipids into tissues. In addition, Li *et al*. [12] suggested that EGCG protects rats from free fatty acid (FFA)-induced insulin resistance.

However, few studies have analysed the inflammatory status of the WAT of HFD-fed mice and the balance of anti- and pro-inflammatory adipokines (IL-10 and TNF-α). In this study, we examined the effects of EGCG consumption on the glycemic and lipid profiles, as well as the expression of TNF-α, adiponectin, IL-10 and NF-κB p65 in the mesenteric WAT of HFD-fed mice.

## Materials and methods

### Animals and treatment

The research committee of Universidade Federal de São Paulo approved all procedures in this study (protocol n° 2009/1796). Three weeks old male Swiss mice were purchased from Centro de Desenvolvimento de Modelos Experimentais para Medicina e Biologia, and kept under controlled conditions of light (12 h light–dark cycle with lights on at 6 am) and temperature (22 ± 1°C). After one week of acclimation, mice were distributed in four groups: 1) NS: normolipidic diet treatment with saline; 2) NE: normolipidic diet treatment with EGCG; 3) HFS: high-fat diet treatment with; 4) HFE: high-fat diet treatment with EGCG.

Throughout the experimental period, the animals were maintained in collective cages and had *ad libitum* access to food and water. The diets were prepared according to the recommendations of the American Institute of Nutrition. The normolipidic groups and the high-fat groups were fed with formulation of the AIN-93G diet for rapid growth [13] (Table 1). A green tea extract, TEAVIGO® (DSM Nutritional Products, Swiss), containing >90% of EGCG (50 mg/kg body weight per day) or saline was administered daily through gavage. Two experimental groups were conducted, one for 30 and another for 60 days.

**Table 1:** **Macronutrients and micronutrients composition of the normolipidic and high-fat diet AIN-93G (g/kg diet)**

Nutrients	Normolipidic diet (g/kg)	High fat diet (g/kg)
**Carbohydrates (g)**	720.7	408.7
**Carbohydrates (kcal)**	75.8%	30.5%
**Protein (g)**	140	140
**Protein (kcal)**	14.7%	10.5%
**Lipids (g)**	40*	352**
**Lipids (kcal)**	9.5%	59%
**Fiber (g)**	50	50
**Vitamin mix (g)**	10	10
**Mineral mix (g)**	35	35
**L-Cysteine (g)**	1.8	1.8
**Choline bitartrate (g)**	2.5	2.5
**Tert-butylhydroquinone (mg)**	14	14
**Energy value**	3.8 kcal/g	5.36 kcal/g

*40 g soybean oil; **312 g lard plus 40 g soybean oil.

### Oral Glucose Tolerance Test (OGTT)

In both experiments (30 or 60 days) all animals were given 12 hours of fast. Initially, the baseline blood was collected to assess basal glucose concentration from the tail vein. Then a glucose solution (1.4 g/kg of body weight) was administrated by gavage. Blood samples were collected again after 15, 30, 45, 60 and 120 minutes to obtain the glycemic curve.

### Sample collection

At the end of each experiment (30 or 60 days), mice were euthanized by decapitation without sedation. Blood was collected and the serum fraction was extracted and stored at -80°C for further analysis. The adipose tissue depots: RET, MES and EPI, were dissected, weighed, immediately frozen in liquid nitrogen and stored at -80°C.

### Lipid profile measurements

Labtest® commercial kits were used to assess serum total cholesterol (TC), HDL-cholesterol and triacylglycerol (TG). The samples were analysed using an enzymatic method.

### Analysis of TNF-α, Adiponectin and IL-10 levels

Adipose tissue samples were carefully rinsed in ice-cold 0.9% NaCl to remove any blood contaminants, snap frozen in liquid nitrogen, and stored at -80°C. Frozen tissue (0.1 g – 0.3 g) was homogenized in RIPA buffer (0.625% Nonidet P-40, 0.625% sodium deoxycholate, 6.25 mM sodium phosphate and 1 mM ethylenediamine tetraacetic acid at pH 7.4) containing 10 mg/ml protease inhibitor cocktail (Sigma–Aldrich, St. Louis, Missouri). Homogenates were centrifuged at 12,000 g for 10 min at 4°C. The supernatant was saved and protein concentration was determined by the Bradford assay (Bio-Rad, Hercules, CA) using bovine serum albumin (BSA) as a standard.

Quantitative assessment of TNF-α, adiponectin and IL-10 levels in adipose tissue was done with ELISA (DuoSet ELISA, R & D Systems, Minneapolis, MN). The TNF-α, adiponectin and IL-10 assay sensitivity was found to be 5.0 pg/ml in the range of 31.2 - 2000 pg/ml. The intra- and inter-assay variability of the TNF-α, adiponectin and IL-10 kits were, respectively, 2.2-4.8%, 3.4-6.7% and 4.9-9.5%. The intra-assay variability of the TNF-α, adiponectin and IL-10 kit was 2.0–4.2%, and its inter-assay variability was of 3.3 -6.4%. All samples were run as duplicates, and the mean value was reported.

### Protein analysis by western blotting

After euthanasia, the MES was dissected and homogenized in 1.0 mL of solubilization buffer at 4°C [1% Triton X-100, 100 mm Tris–HCl (pH 7.4), 100 mm sodium pyrophosphate, 100 mm sodium fluoride, 10 mm EDTA, 10 mm sodium orthovanadate, 2.0 mm phenylmethylsulfonyl fluoride (PMSF), and 0.1 mg aprotinin/mL] with a Polytron (model 713 T; Fisatom Equipamentos Científicos, São Paulo, SP/Brazil). Insoluble material was removed by centrifugation for 30 min at 9,000 × *g* in a 70.Ti rotor (Beckman, Fullerton, CA, USA) at 4°C. The protein concentration of the supernatants was performed by the BCA assay (Bio-Rad, Hercules, CA, USA). Proteins were denatured by boiling (5 min) in a Laemmli sample buffer ^10^ containing 100 mM DTT, run on 8, 10 or 12% SDS-PAGE in a Bio-Rad miniature slab gel apparatus.

The electrotransfer of proteins from gels to nitrocellulose membranes was performed for ~1.30 h/4gels at 15 V (constant) in a Bio-Rad semi-dry transfer apparatus. Nonspecific protein binding to the nitrocellulose was reduced by preincubation for 2 h at 22°C in blocking buffer (5% nonfat dry milk, 10 mM Tris, 150 mM NaCl and 0.02% Tween 20). The nitrocellulose membranes were incubated overnight at 4°C with antibodies against NFκBp65 and alpha-tubulin obtained from Santa Cruz Biotechnology (Santa Cruz, CA, USA), diluted in 1:1000 with blocking buffer supplemented with 1% BSA and then washed for 30 min in blocking buffer without BSA. The blots were subsequently incubated with peroxidase-conjugated secondary antibody for 1 h at 22°C. For evaluation of protein loading, membranes were stripped and reblotted with an anti-alpha-tubulin antibody as appropriate. Specific bands were detected by chemiluminescence and visualization/capture was performed by exposure of the membranes to RX films. B and intensities were quantified by optical densitometry of developed autoradiographs (Scion Image software-Scion Corporation, Frederick, Md., USA).

### Statistical analysis

All results are presented as means ± standard error of the mean (SEM). Statistical significances were assessed using two-way analysis of variance (ANOVA) followed by Tukey test as a post hoc analysis to identify significant differences among the groups. Differences were considered significant when p < 0.05.

## Results

### Body mass and tissue weight

Significant differences in body mass and tissue weight were found only in the 60-day treatment groups. Differences in the delta weight (g) were observed between the HFS and NS groups (p < 0.0001), the HFE and NE groups (p = 0.042) and the HFS group (p = 0.0036).

The relative weight of the EPI in the HFS group was significantly higher than the NS group (p < 0.0001) and the HFE group was significantly higher than the HFS group (p = 0.0098).

The relative weights of RET, MES and SAT were higher in the HFS group compared to the NS and HFE groups and the HFE was higher than the NE group (p ≤ 0.02) (Table 2).

**Table 2:** **Body and adipose tissue weights during two treatment periods (30 days or 60 days) in the experimental groups**

Parameters	30 days of treatment (n = 5-7 per group)				60 days of treatment (n = 11-13 per group)			
	N S	NE	HFS	HFE	N S	NE	HFS	HFE
**Initial weight (g)**	31.7 ± 0.8	32.9 ± 1.1	31.5 ± 0.8	29.8 ± 1.1	26.7 ± 0.8	26.4 ± 0.5	28.9 ± 0.8	24.7 ± 1.0
**Final weight (g)**	37.8 ± 3.1	34.8 ± 5.7	36.7 ± 5.6	37.7 ± 6.0	32.7 ± 1.0	31.4 ± 0.8	42.5 ± 1.2*	33.8 ± 1.8^$^
**Delta weight (g)**	6.2 ± 1.3	3.3 ± 1.4	5.8 ± 1.3	8.6 ± 1.4	6.1 ± 0.8	5.0 ± 0.8	13.7 ± 1.5*	9.1 ± 1.0**^$^
**EPI (g)**	1.49 ± 0.2	1.15 ± 0.2	1.52 ± 0.2	1.67 ± 0.3	0.8 ± 0.1	0.7 ± 0.1	2.0 ± 0.2*	1.0 ± 0.2^$^
**EPI (%)**	3.80 ± 0.7	3.11 ± 0.7	3.82 ± 0.7	4.05 ± 0.9	2.5 ± 0.3	2.2 ± 0.2	4.6 ± 0.4*	3.2 ± 0.3^$^
**RET (g)**	0.38 ± 0.1	0.35 ± 0.1	0.47 ± 0.1	0.43 ± 0.1	0.3 ± 0.04	0.2 ± 0.02	0.6 ± 0.1*	0.4 ± 0.1^$^
**RET (%)**	0.96 ± 0.2	0.98 ± 0.2	1.19 ± 0.2	1.07 ± 0.2	0.8 ± 0.1	0.6 ± 0.1	1.4 ± 0.1*	1.1 ± 0.1**^$^
**MES (g)**	0.55 ± 0.1	0.51 ± 0.1	0.60 ± 0.1	0.68 ± 0.1	0.3 ± 0.05	0.2 ± 0.03	0.8 ± 0.1*	0.4 ± 0.1^$^
**MES (%)**	1.39 ± 0.2	1.42 ± 0.3	1.51 ± 0.2	1.67 ± 0.3	0.8 ± 0.1	0.8 ± 0.1	1.9 ± 0.2*	1.4 ± 0.2**^$^
**SAT (g)**	2.41 ± 0.3	2.02 ± 0.3	2.60 ± 0.3	2.78 ± 0.5	1.3 ± 0.2	1.1 ± 0.1	3.5 ± 0.3*	1.8 ± 0.2^$^
**SAT (%)**	6.16 ± 0.7	5.51 ± 0.5	6.53 ± 0.6	6.79 ± 0.9	3.2 ± 0.6	3.6 ± 0.3	7.4 ± 0.8*	4.4 ± 0.7**^$^

Data are mean ± SEM. *Different from NS. **Different from NE. ^$^Different from HFS.

### Biochemical and hormonal serum analyses

During the 30-day treatment period, only the TC values differ between the groups, in the HFS group were lower than in the NS group (p =0.016), and the HFE group had a higher concentration compared to the HFS group (p < 0.001).

On the other hand, during the 60-day treatment period, we observed that TG in the HFE group was significantly lower than in the NE group (p = 0.047). There were no differences in TC between groups. HDL levels were lower in the HFS group compared to the NS group (p = 0.0031) and higher in the HFE group compared to the HFS group (p = 0.0012). There was a significant decrease in serum levels of insulin in the HFE group compared to the HFS group (p = 0.022).

We observed that adiponectin in the NS group increased in comparison to the NE (p = 0.008) and HFS groups (p < 0.0001) and that adiponectin in the HFE group decreased in comparison to the NE group (p = 0.009). In addition, the adiponectin/SAT ratio was lower in the HFS group comparison to the NS group (p = 0.001) and higher in the HFE group comparison to the HFS group (p = 0.009) (Table 3).

**Table 3:** **Triacylglycerol (TG), Total Cholesterol (TC), High Density Lipoprotein - Cholesterol (HDL- C), insulin, adiponectin, adiponectin /sum of adipose tissue (SAT) concentrations during two treatment periods (30 days our 60 days) in the experimental groups**

Parameters	30 Days of Treatment (n = 5-7 per group)				60 Days of Treatment (n = 11-13 per group)			
	N S	NE	HFS	HFE	N S	NE	HFS	HFE
**TG (mg/dL)**	169.8 ± 19.1	166.8 ± 9.9	152.0 ± 8.5	137.6 ± 5.3	174.0 ± 16.1	184.0 ± 14.4	145.1 ± 5.4	136.6 ± 5.4**
**TC (mg/dL)**	174.7 ± 7.5	176.5 ± 5.0	155.7 ± 7.5*	181.6 ± 6.5^$^	176.7 ± 6.4	212.0 ± 11.4	194.9 ± 13.6	178.1 ± 8.6
**HDL-C (mg/dL)**	36.21 ± 2.2	40.99 ± 4.8	39.32 ± 4.5	36.89 ± 1.9	42.1 ± 1.3	44.4 ± 0.8	37.0 ± 1.0*	42.6 ± 1.7^$^
**Insulin (ng/mL)**	0.6 ± 0.1	0.5 ± 0.1	0.3 ± 0.04	0.4 ± 0.05	1.2 ± 0.2	1.0 ± 0.3	1.9 ± 0.2	0.8 ± 0.1^$^
**Adiponectin (μg/mL)**	-	-	-	-	3.4 ± 0.2	2.5 ± 0.2*	1.8 ± 0.2*	1.6 ± 0.1**
**Adiponectin/SAT**	-	-	-	-	99.8 ± 14.8	91.6 ± 8.6	30.1 ± 5.4*	33.9 ± 5.2^$^

Data are mean ± SEM (n = 5-8 per group); *Different from NS. **Different from NE. ^$^Different from HFS.

### Cytokines in mesenteric adipose tissue

In both treatment periods (30 or 60 days), no differences were observed in mesenteric cytokines levels between any of the groups (data not shown).

### OGTT

**Figure 1:**
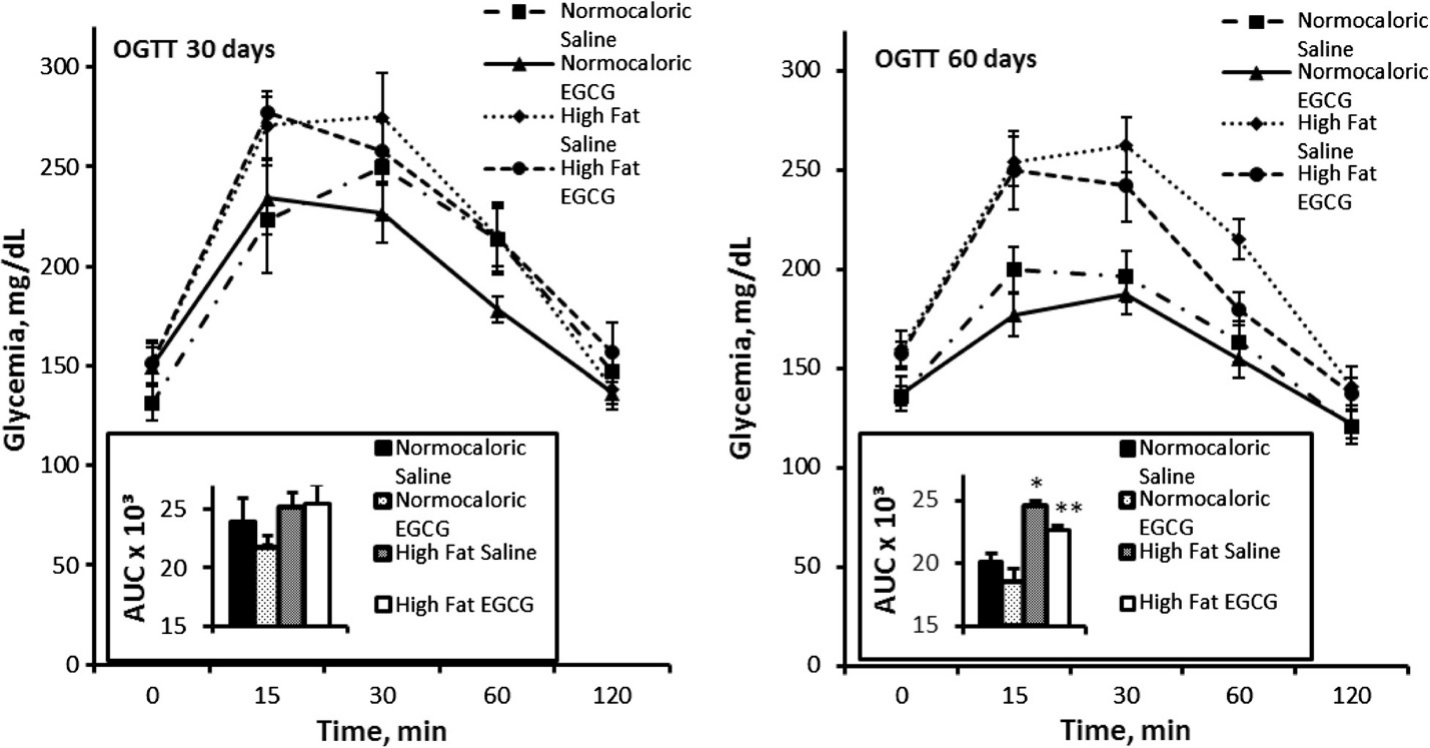
**Oral Glucose Tolerance Test (OGTT) during two treatment periods (30 days our 60 days) in the experimental groups.** *Different from NS (p = 0.003); ** different from HFE (p = 0.001).

Glucose tolerance, as measured by OGTT, did not differ between any of the groups in the 30-day treatment period. However, in the 60-day treatment groups, we observed differences between the HFS group and the NS group (p = 0.003) as well as between the HFE group and the NE group (p = 0.001) (Figure 1).

### Quantification of inflammatory proteins

Western blot analysis shows that there were no differences in NF-kBp65 protein levels between any of the groups treated for 30 days.

**Figure 2:**
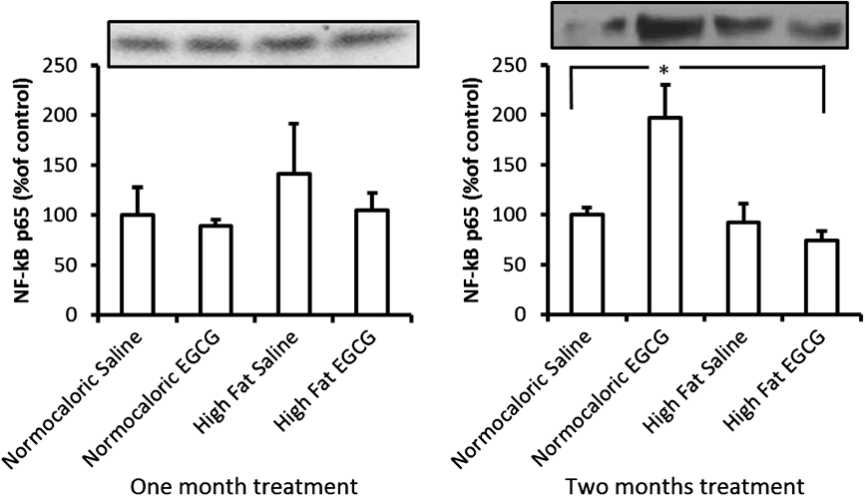
**Quantification of NF-kB p65 protein levels during the two treatment periods (30 days our 60 days) in the experimental groups.** *Different from NS compared to the others groups (p = 0.005).

However, NF-kB p65 expression in the MES was increased in the NE group compared to the other groups after the 60-day treatment period (p < 0.005) (Figure 2).

## Discussion

The pathogenesis of obesity and metabolic diseases is associated with intake of a high-fat diet. Chronic systemic inflammation directly contributes to the development of obesity [14]. Therefore, suppressing chronic inflammation may be a good strategy to prevent and/or treat obesity. Interestingly, previous studies suggest that the positive impacts of polyphenols could work through their ability to suppress chronic inflammation [15, 16]. Based on this knowledge, an alternative strategy, such as phytotherapy treatment, may benefit modern obesity therapies.

In this experimental study, we attempted to induce metabolic changes in mice by administration of a high-fat diet and then compared the outcomes of treatment with EGCG or saline over either 30-day or 60-day periods. Our results show that the treatment of *Swiss* mice with a high-fat diet for 30 days did not increase weight gain, relative tissue weights or induce significant changes in OGTT or inflammatory profile. The experimental period of 30 days may be too short to show significant changes in these parameters.

On the other hand, we demonstrated that a 60-day treatment period of high-fat diet and EGCG treatment was effective in triggering inflammatory processes such as a decrease in adiponectin and induced changes in glucose homeostasis, weight mass, TG and HDL-C.

The anti-obesity effects of green tea are most likely due to its capacity to elevate thermogenesis and fat oxidation [17]. Thus, we hypothesised that EGCG treatment reduces body-fat mass by changes in cytokine production and pro-inflammatory molecule protein levels. Our study is in agreement with other reports in the literature that clinical studies have shown that the consumption of EGCG is connected with weight loss [18].

Our results contrast with those currently expected once the literature evidences the increase in adiponectin with weight loss [19]. However, some studies also showed no increase in adiponectin with weight loss but still found an improvement in insulin resistance, agreeing with the findings of this study [20, 21]. It is possible that weight loss can lead to increased insulin sensitivity in other ways independent of the action of adiponectin, such as mobilisation of intracellular lipid content of the liver [22].

When we analysed serum adiponectin levels we observed that the high-fat diet was effective in reducing serum adiponectin levels and that EGCG contributed to the decrease in both, normolipidic as in high-fat diet. However, when we need to consider the amount of fat in each mouse and adjust the serum adiponectin level with the SAT, the results showed that EGCG improved the adiponectin concentration.

In another study, green tea extract added to the diet (1%) did not alter the concentrations of adiponectin [23] in adult mice fed a high-fat diet for 12 weeks. Addition of 1.2% green tea extract into the water decreased adiponectin concentrations in mice after eight weeks of treatment; however [24]. By adjusting our results for adiponectin/STA we observed that the negative effect of green tea disappears. The change in adiponectin could be explained by the reduced fat mass in the HFS and HFE groups since this is a cytokine that is produced by adipocytes.

EGCG treatment caused decreased adipose tissue deposits corresponding to the improvement of insulin levels in the HFE group. Several studies have shown that weight loss is associated with decreased insulin resistance and inflammatory markers [25]. These findings demonstrate that EGCG may be a novel, plant-derived compound capable of reducing the risk of HFD-induced glucose intolerance.

Fu et al. [26] examined the potential (-)-epigallocatechin gallate (EGCG, 0.05% in drinking water) on effectively delaying the onset of type 1 diabetes (T1D) in non-obese diabetic (NOD) mice. Mice supplemented with EGCG had significantly higher plasma insulin levels and survival rates compared with the control animals. EGCG had no significant effects on food or water intake or body weight in mice, suggesting that the glucose-lowering effect was not due to an alteration in these parameters. While EGCG did not modulate insulinemia, it did elevate the levels of the circulating anti-inflammatory cytokine IL-10 in NOD mice. These findings demonstrate that EGCG may be a novel, plant-derived compound capable of reducing the risk of T1D.

Obesity and insulin resistance are associated with low grade chronic systemic inflammation [27]. In the present study, we observed no changes in the cytokine profile during either treatment period (30 days or 60 days). However, we did observe differences in delta weight, relative RET, MES, EPI and SAT weights, adiponectin levels and glucose homeostasis. We hypothesise that these differences will become more pronounced in a longer trial period (>60 days).

We see the normocaloric diet with EGCG increased phosphorylation of the p65 subunit from the NF-kB complex in MES. These are unexpected effects of the administration of EGCG. Perhaps the consumption of antioxidants from the EGCG caused an adverse effect on the NE group.

In summary, our data demonstrated that the administration of EGCG (60 days) promoted a significant improvement in glucose tolerance, decreased adipose tissue deposits, weight mass, TG and HDL-C only when associated with high-fat diet treatment. More studies are required to better understand the mechanism of this effect and to further elucidate the role of EGCG in reversing the inflammatory effects triggered by a high-fat diet.

## Abbreviations

HFDs: High-fat diets
EGCG: Epigallocatechin-3-gallate
NS: Normolipidic diet treatment with saline
NE: Normolipidic diet treatment with EGCG
HFS: High-fat diet treatment with
HFE: High-fat diet treatment with EGCG
TNF-α: Tumor necrosis factor alpha
ELISA: Enzyme-linked immunosorbent assay
NF-κB: Nuclear factor kappa B
WAT: White adipose tissue
MES: Mesenteric adipose tissue
RET: Retroperitonial adipose tissue
SAT: Sum of adipose tissues
TG: Triacylglycerol
FFA: Free fatty acid
TC: Total cholesterol
HDL- C: High density lipoprotein - Cholesterol.

## References

[1] Jequier E (2002). Pathways to obesity. Int J Obes Relat Metab Disord.

[2] Yamashita AS, Lira FS, Rosa JC, Paulino EC, Brum PC, Negrao CE, dos Santos RV, Batista ML, do Nascimento CO, Oyama LM, Seelaender M (2010). Depot-specific modulation of adipokine levels in rat adipose tissue by diet-induced obesity: the effect of aerobic training and energy restriction. Cytokine.

[3] Nascimento CM O d, Ribeiro EB, Oyama LM (2009). Metabolism and secretory function of white adipose tissue: effect of dietary fat. An Acad Bras Cienc.

[4] Trayhurn P (2005). Endocrine and signalling role of adipose tissue: new perspectives on fat. Acta Physiol Scand.

[5] Lira FS, Rosa JC, Cunha CA, Ribeiro EB, do Nascimento CO, Oyama LM, Mota JF (2011). Supplementing alpha-tocopherol (vitamin E) and vitamin D3 in high fat diet decrease IL-6 production in murine epididymal adipose tissue and 3 T3-L1 adipocytes following LPS stimulation. Lipids Health Dis.

[6] Sae-Tan S, Grove KA, Kennett MJ, Lambert JD (2011). (-)-Epigallocatechin-3-gallate increases the expression of genes related to fat oxidation in the skeletal muscle of high fat-fed mice. Food function.

[7] Klaus S, Pultz S, Thone-Reineke C, Wolfram S (2005). Epigallocatechin gallate attenuates diet-induced obesity in mice by decreasing energy absorption and increasing fat oxidation. Int J Obes (Lond).

[8] Thielecke F, Boschmann M (2009). The potential role of green tea catechins in the prevention of the metabolic syndrome - a review. Phytochemistry.

[9] Yang CS, Wang X, Lu G, Picinich SC (2009). Cancer prevention by tea: animal studies, molecular mechanisms and human relevance. Nat Rev Cancer.

[10] Venables MC, Hulston CJ, Cox HR, Jeukendrup AE (2008). Green tea extract ingestion, fat oxidation, and glucose tolerance in healthy humans. Am J Clin Nutr.

[11] Friedrich M, Petzke KJ, Raederstorff D, Wolfram S, Klaus S (2012). Acute effects of epigallocatechin gallate from green tea on oxidation and tissue incorporation of dietary lipids in mice fed a high-fat diet. Int J Obes (Lond).

[12] Li Y, Zhao S, Zhang W, Zhao P, He B, Wu N, Han P (2011). Epigallocatechin-3-O-gallate (EGCG) attenuates FFAs-induced peripheral insulin resistance through AMPK pathway and insulin signaling pathway in vivo. Diabetes Res Clin Pract.

[13] Reeves PG (1997). Components of the AIN-93 diets as improvements in the AIN-76A diet. J Nutr.

[14] Rankinen T, Zuberi A, Chagnon YC, Weisnagel SJ, Argyropoulos G, Walts B, Perusse L, Bouchard C (2006). The human obesity gene map: the 2005 update. Obesity (Silver Spring).

[15] Shen CL, Chyu MC, Pence BC, Yeh JK, Zhang Y, Felton CK, Doctolero S, Wang JS (2010). Green tea polyphenols supplementation and Tai Chi exercise for postmenopausal osteopenic women: safety and quality of life report. BMC Compl Alternative Med.

[16] Shen CL, Yeh JK, Samathanam C, Cao JJ, Stoecker BJ, Dagda RY, Chyu MC, Dunn DM, Wang JS (2011). Green tea polyphenols attenuate deterioration of bone microarchitecture in female rats with systemic chronic inflammation. Osteoporos Int.

[17] Boschmann M, Thielecke F (2007). The effects of epigallocatechin-3-gallate on thermogenesis and fat oxidation in obese men: a pilot study. J Am Coll Nutr.

[18] Lonac MC, Richards JC, Schweder MM, Johnson TK, Bell C (2011). Influence of short-term consumption of the caffeine-free, epigallocatechin-3-gallate supplement, Teavigo, on resting metabolism and the thermic effect of feeding. Obesity (Silver Spring).

[19] Esposito K, Nappo F, Giugliano F, Di Palo C, Ciotola M, Barbieri M, Paolisso G, Giugliano D (2003). Meal modulation of circulating interleukin 18 and adiponectin concentrations in healthy subjects and in patients with type 2 diabetes mellitus. Am J Clin Nutr.

[20] Xydakis AM, Case CC, Jones PH, Hoogeveen RC, Liu MY, Smith EO, Nelson KW, Ballantyne CM (2004). Adiponectin, inflammation, and the expression of the metabolic syndrome in obese individuals: the impact of rapid weight loss through caloric restriction. J Clin Endocrinol Metab.

[21] Marcell TJ, McAuley KA, Traustadottir T, Reaven PD (2005). Exercise training is not associated with improved levels of C-reactive protein or adiponectin. Metabolism.

[22] Tiikkainen M, Bergholm R, Vehkavaara S, Rissanen A, Hakkinen AM, Tamminen M, Teramo K, Yki-Jarvinen H (2003). Effects of identical weight loss on body composition and features of insulin resistance in obese women with high and low liver fat content. Diabetes.

[23] Shirai N, Suzuki H (2008). Effects of simultaneous intakes of fish oil and green tea extracts on plasma, glucose, insulin, C-peptide, and adiponectin and on liver lipid concentrations in mice fed low- and high-fat diets. Ann Nutr Metab.

[24] Zhou JR, Li L, Pan W (2007). Dietary soy and tea combinations for prevention of breast and prostate cancers by targeting metabolic syndrome elements in mice. Am J Clin Nutr.

[25] Nicklas BJ, You T, Pahor M (2005). Behavioural treatments for chronic systemic inflammation: effects of dietary weight loss and exercise training. CMAJ.

[26] Fu Z, Zhen W, Yuskavage J, Liu D (2011). Epigallocatechin gallate delays the onset of type 1 diabetes in spontaneous non-obese diabetic mice. Br J Nutr.

[27] Wellen KE, Hotamisligil GS (2005). Inflammation, stress, and diabetes. J Clin Invest.

